# Statistical Analysis of the Random Telegraph Noise in a 1.1 μm Pixel, 8.3 MP CMOS Image Sensor Using On-Chip Time Constant Extraction Method †

**DOI:** 10.3390/s17122704

**Published:** 2017-11-23

**Authors:** Calvin Yi-Ping Chao, Honyih Tu, Thomas Meng-Hsiu Wu, Kuo-Yu Chou, Shang-Fu Yeh, Chin Yin, Chih-Lin Lee

**Affiliations:** Taiwan Semiconductor Manufacturing Company, Hsinchu Science Park, Hsinchu 300, Taiwan; hytu@tsmc.com (H.T.); mhwuzd@tsmc.com (T.M.-H.W.); kychouc@tsmc.com (K.-Y.C.); sfyehe@tsmc.com (S.-F.Y.); cyin@tsmc.com (C.Y.); clleeza@tsmc.com (C.-L.L.)

**Keywords:** CMOS image sensor (CIS), random telegraph noise (RTN), correlated double sampling (CDS), RTN emission and capture time constant, backside-illuminated technology (BSI), pinned photodiode (PPD), Gumbel distribution

## Abstract

A study of the random telegraph noise (RTN) of a 1.1 μm pitch, 8.3 Mpixel CMOS image sensor (CIS) fabricated in a 45 nm backside-illumination (BSI) technology is presented in this paper. A noise decomposition scheme is used to pinpoint the noise source. The long tail of the random noise (RN) distribution is directly linked to the RTN from the pixel source follower (SF). The full 8.3 Mpixels are classified into four categories according to the observed RTN histogram peaks. A theoretical formula describing the RTN as a function of the time difference between the two phases of the correlated double sampling (CDS) is derived and validated by measured data. An on-chip time constant extraction method is developed and applied to the RTN analysis. The effects of readout circuit bandwidth on the settling ratios of the RTN histograms are investigated and successfully accounted for in a simulation using a RTN behavior model.

## 1. Introduction

Random telegraph noise (RTN) is a type of low-frequency temporal noise encountered in many semiconductor devices [1,2,3,4,5,6,7]. In CMOS image sensors (CIS) and charge coupled devices (CCD), the RTN is mainly responsible for the blinking and twinkling pixels [8,9] in the videos captured under low light conditions. The RTN causes unpleasant visual artifacts, decreases the sensor dynamic range, and degrades the image quality.

The RTN also has adverse impact on the performance of a broad range of products such as the DRAM [10,11], the SRAM [12,13], the NAND Flash [14,15], as well as the generic analog and digital circuits [16,17]. As the trend of technology scaling continues, the RTN has the potential of exceeding all the other random process variations as the major source of device variability and the concern of reliability in advanced nodes [18,19].

It is not an overstatement that RTN was called the circuit designer’s nightmare [6], partly because it is difficult to model and to incorporate RTN in the circuit simulation, either in the time or the frequency domain. The RTN distribution is typically highly skewed and non-Gaussian with many possible numbers of traps and a wide range of time constants, making it difficult to specify a worst-case process corner for design sign-off. For the array type of devices, such as memories and imager sensors, the percentage of cells showing clear RTN behavior may not be very high, but the impact on the yield, the error rates, and the image quality cannot be ignored. In contrast, the thermal noises, the flicker noises, and other process variations are generally included and supported in the simulation models with well-defined corners.

The sources of the RTN are believed to be the charge traps related to defects inside the gate oxide, at the oxide–silicon interface, or at the STI–silicon interface. To fully understand the RTN, it is necessary to characterize the statistical distributions, emission-capture time constants, activation energies, noise power spectra densities, physical locations, and the relations to detailed fabrication processes. In spite of being studied in the past several decades [20,21,22,23,24,25,26,27,28,29,30,31,32,33,34,35,36,37,38,39,40,41,42,43,44,45,46], many RTN-related issues still remain as active research topics due to the nature of its complexity.

In this work, we focus on the RTN pixel classification and the time constant extraction in CMOS image sensors. Most of the RTN time constant extraction reported in the literature [20,21,22,23,24,25,26,27,28,29,30,31,32,33,34,35,36,37,38,39,40,41,42,43,44,45,46] was based on the telegraphic waveforms of measured voltages or currents. Here, we present a new method of on-chip time constant extraction based on double sampling circuits. From the noise dependence on the time difference between the two phases of the double sampling, the time constants for a large number of pixels can be extracted efficiently.

In the following, the details of the CIS test chip are described in Section 2. The identification of the noise source is demonstrated in Section 3. The RTN distribution and RTN pixel classification are summarized in Section 4. The theoretical equation underlying the time constant extraction and the pixel-by-pixel time constant extraction results are presented in Section 5. The effects of readout circuit bandwidth and settling time are analyzed in Section 6. The temperature dependence of the random noise (RN) medians, the number of RTN pixels, and the time constant distributions are discussed in Section 7. Some of the unresolved issues are pointed out in Section 8. Finally, the conclusions are given in Section 9. A subset of these results was published in [44,45].

## 2. The 8.3MP, 1.1 μm Pixel Backside-Illuminated CIS

**Figure 1:**
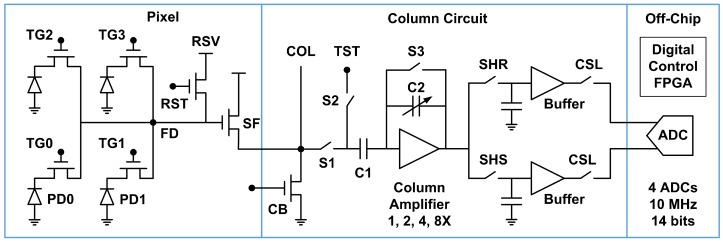
A simplified schematic of the signal chain. The 3296 × 2512 pixel array consists of 1648 × 1256 groups of 2 × 2, 4-shared pixels. Four off-chip 14 bits ADCs digitize the buffered analog voltages and the off-chip FPGA combines and outputs the digital data at 10 MHz clock. The even column pixels and the odd column pixels are read out in the first and the second half line time.

In this paper, we study the temporal RN, especially the RTN, of an 8.3 Mpixel CIS test chip fabricated in a 45 nm backside illumination (BSI) CIS technology. The pixel has a 1.1 μm pitch and a 2 × 2-shared structure, with an average of 1.5 transistors per cell. Figure 1 is a simplified signal chain schematic. The column circuit consists of a switched-capacitor amplifier with a programmable gain from 1X to 8X, a pair of sample-and-hold (S/H) circuits for correlated double sampling (CDS), the capacitor buffers, and the column-select (CSL) switches. The differential CDS outputs are digitized by four off-chip 14-bit ADCs with a 2 V differential input range. The timing generation, rolling shutter control, and digital processing are implemented in an on-board FPGA, which combines, reformats, and outputs the digital data at 10 MHz.

The pinned photodiode (PPD) [47] in each pixel has an average full-well capacity (FWC) around 6000 e−. The pixel source follower (SF) is a low-Vth (~0.2 V) 3.3 V NMOS with W = 0.2 μm, L = 0.8 μm, and a nominal gate oxide thickness of 5.6 nm. The average SF gain is 0.85 when biased at a 4 μA constant current. The system conversion factors measured by the photon transfer curve (PTC) method [48] are 1.24 e−/DN, 0.61 e−/DN, 0.315 e−/DN, and 0.153 e−/DN at 1X, 2X, 4X, and 8X gain, respectively. The estimated pixel floating diffusion (FD) capacitance is 1.34 fF and the corresponding conversion gain is 120 μV/e−. The physical and performance parameters of the chip are summarized in Table 1.

**Figure 2:**
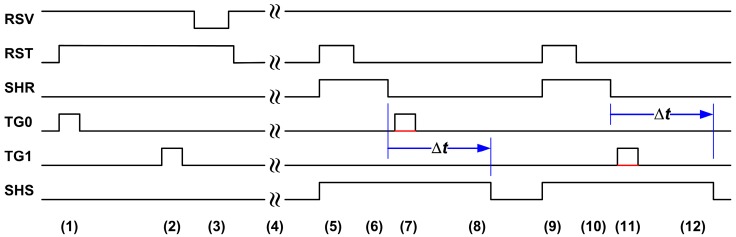
A simplified timing control diagram for the following sequence of operations: (1) even pixel PD reset; (2) odd pixel PD reset; (3) SF turned off; (4) exposure and charge integration; (5) FD reset; (6) reset noise sampling; (7) even pixel charge transfer; (8) even pixel photo signal sampling; (9) FD reset; (10) reset noise sampling; (11) odd pixel charge transfer; (12) odd pixel photo signal sampling. During the dark measurement, the transfer gate pulses TG0 (even) and TG1 (odd) may be disabled and the time difference Δ*t* between the first sampling (SHR) and the second sampling (SHS) can be programmed from 0 to 25 μs by 0.1 μs steps.

Figure 2 is a simplified operation timing diagram. Because the even- and odd-column pixels share the same vertical column line, they are read out alternatively in the first and the second half-line time. The 2 half-line digital data are re-assembled in the FPGA to form a complete line output. The normal operation consists of the following sequential steps: (1) even-column pixel PD reset; (2) odd-column pixel PD reset; (3) SF turned off; (4) exposure and charge integration; (5) FD reset; (6) reset noise sampling; (7) even-column pixel charge transfer; (8) even-column pixel photo signal sampling; (9) FD reset; (10) reset noise sampling; (11) odd-column pixel charge transfer; (12) odd-column pixel photo signal sampling.

During the dark measurement, the transfer gate pulses TG0 and TG1 may be disabled and the time difference Δ*t* between the first and the second sampling can be programmed from 0 to 25 μs by 0.1 μs steps. This particular feature provides us with a means to observe the SF RTN in a transition from the correlated to the uncorrelated double sampling, and forms the basis of on-chip RTN time constant extraction, to be discussed in Section 5.

## 3. Noise Source Decomposition Scheme

There are two types of noise in CIS, the time-domain temporal noise and the spatial-domain fixed-pattern noise (FPN). In this study, we are only concerned with the temporal noise. The major temporal noise sources in this design are listed in Table 2. In a typical operation under light, the photon shot noises are the dominant temporal noises. When the measurements are done in the dark, the photon shot noises can be ignored. For small pixels, the KTC reset noises, if not properly cancelled, would be significantly high due to the small FD capacitance. Due to the PPD technology and the CDS technique [48], the reset noises can be cancelled almost completely.

The reset noises, once sampled and stored on the FD, remain unchanged from the first to the second sampling. Therefore, the double sampling is correlated for the reset noises. However, this is not the case for the SF, the column bias, or the column amplifier noises. As a matter of fact, the doubling sampling is essentially uncorrelated with respect to the SF, the column bias, and column amplifier noises because these noises are time dependent and there is a time difference Δ*t* between the first and the second sampling.

This chip has two important designed-in features for us to study the noise composition. First, the double sampling Δ*t* can be programed. When Δ*t* is 0, the noises from the SF, the column bias, or the column amplifier are all cancelled; but they are not when Δ*t* is nonzero. Second, the pixels can be bypassed by opening the S1 switch and closing the S2 switch. An external signal can be injected through the test port TST. Combining the above “pixel-in” and “test-in” with the “Δ*t* = 25 μs” and “Δ*t* = 0 μs”, we could characterize the noises in four operation modes as shown in Table 2.

**Figure 3:**
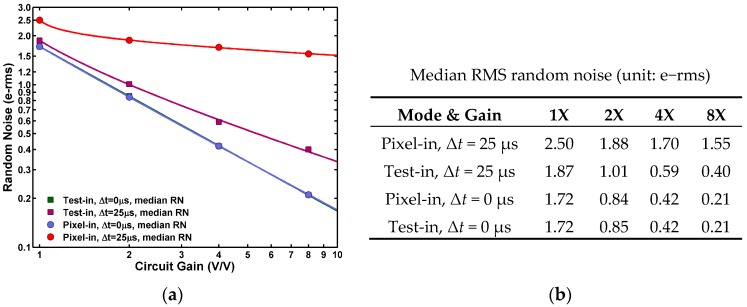
(**a**) The measure median RN for four-operation modes under 1X, 2X, 4X, and 8X analog gains. (**b**) The tabulated median RN of four-operation modes corresponding to the graph in (a).

Figure 3 shows the measured median noises of the 8.3 M array under 1X, 2X, 4X, and 8X analog gains. The noises are calculated as the RMS of 5000 frames, pixel by pixel, and input-referred to the FD in the unit of e−rms. As can be seen, the rest of the circuits contribute as little as 0.2 e−rms at 8X gain.

The noise is increased to 0.4 e−rms if the column amplifiers are included. The noise is further increased to 1.55 e−rms when the SF and the column bias noises are included. From the noise power point of view, clearly the SF and the column bias dominate.

**Figure 4:**
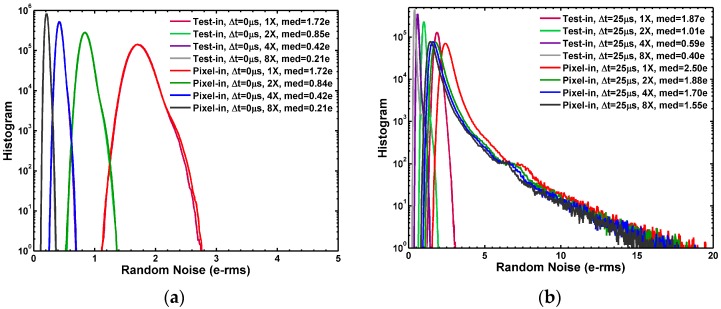
(**a**) The RN histogram measured at 1X, 2X, 4X, and 8X gains for the “test-in, Δ*t* = 0 μs” and “pixel-in, Δ*t* = 0 μs” modes. The distributions are tight Gaussian-like without noticeable long tails; (**b**) The RN histograms measured at 1X, 2X, 4X, and 8X gains for the “test-in, Δ*t* = 25 μs” and “pixel-in, Δ*t* = 25 μs” modes. Among the 4 operation modes, only the histograms of the “pixel-in, Δ*t* = 25 μs” mode show significant long tails.

It is not sufficient to just look at the medians of the noises; it is more interesting to examine the statistical distribution of the noises. Figure 4a,b show that, for three out of the four configurations in which the SF and the column bias are excluded, the noise histograms are tight and close to the standard Gaussian distribution without any noticeable long tails. On the contrary, in the case of “pixel-in, Δ*t* = 25 μs” where the SF and the column bias are included, the histogram shows a very prominent long tail. Even though the median RN is only 1.55 e−rms under 8X gain and the RN in the tail region could be as high as 27 e−rms. This long tail distribution is generally attributed to the RTN from the SF, which is the central topic of the following discussion.

**Figure 5:**
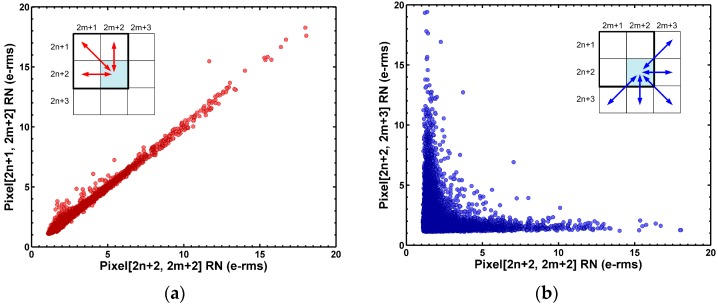
(**a**) For the 2 × 2, shared pixel structure, each pixel shares the same SF with three adjacent neighbors, and does not share the same SF with the other five adjacent neighbors. The random noises among pixels sharing the same SF are highly correlated; (**b**) On the contrary, the random noises among pixels not sharing the same SF are not correlated. This is indirect evidence that SF is the dominant noise source.

For a 2 × 2 pixel structure, each pixel shares the same SF with three adjacent neighbors, but do not share the SF with the other five adjacent pixels. In the scatter plots of Figure 5, we systematically verified that the noises among the pixels sharing the same SF are highly correlated.

In contrast, the noises among the pixels not sharing the same SF are not correlated at all. Again, this is an indirect but convincing proof that the SF is indeed the main source of the histogram tail.

## 4. RTN Pixel Classification

**Figure 6:**
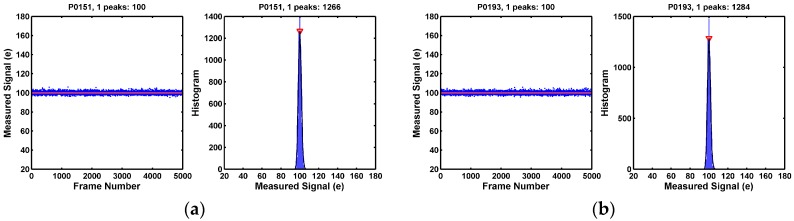
(**a**,**b**) Two example pixels showing one histogram peak with small RMS noises. These pixels do not appear to have any RTN traps. They account for a majority of the 8.3 Mpixels, more than 95% of the population, on the lower noise end of the distribution. A set of selected 1000 pixels are labelled from “P0001” to “P1000”.

To investigate the nature of the noises in the long tail, we inspect each individual pixel’s signal fluctuation over 5000 frames and its corresponding distribution. The goal is to categorize the pixels according to their time domain behaviors. For most of the low-noise pixels, the signals are tightly distributed and the histogram is approximately Gaussian-like, as shown in Figure 6.

**Figure 7:**
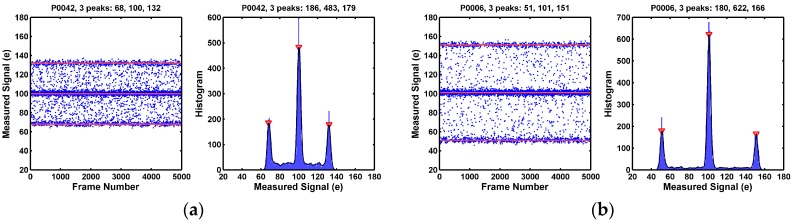
(**a**,**b**) Two example pixels showing 3 clearly identifiable histogram peaks. This is the signature behavior of pixels with a single RTN trap as the result of the CDS subtraction. The 2 side peaks are symmetric in magnitudes and in populations, which indicates that the probabilities of trap occupancy (PTO) during the first sampling and the second sampling are approximately equal.

On the other hand, as illustrated in Figure 7, many of the pixels in the long tail portion of the noise distribution show distinctively 3 discrete peaks in the histograms. This is the signature behavior of a single trap RTN under the CDS operation. A single electron trap in the SF may be empty or occupied. A trapped electron causes an equivalent shift of the SF threshold voltage by +Δ*V*, causing the SF output shift by −Δ*V*. There are 4 possible combinations of the trap states during the double sampling: (empty, empty), (empty, occupied), (occupied, empty), and (occupied, occupied). These lead to 3 discrete levels (−Δ*V*, 0, +Δ*V*) after the CDS subtraction.

**Figure 8:**
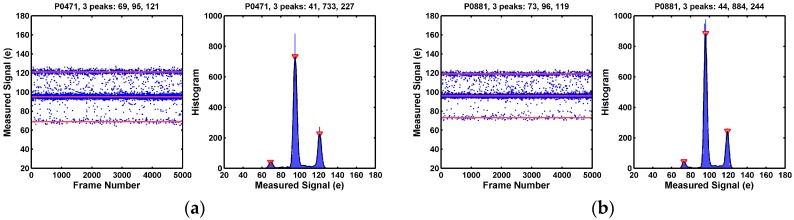
(**a**,**b**) Two example pixels showing 3 clearly identifiable histogram peaks with 2 asymmetric side peaks. These are believed to be the single RTN trap behaviors as well. But the probabilities of trap occupancy (PTO) during the first sampling and the second sampling of the CDS are not equal. The mechanism causing the asymmetry is discussed in Section 8.1.

**Figure 9:**
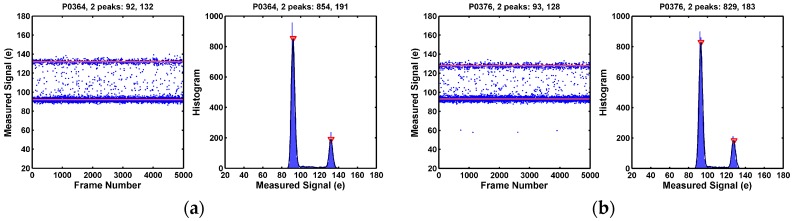
(**a**,**b**) Two example pixels showing 2 identifiable histogram peaks, which are the degenerated cases of the 3-peak pixels. These pixels are considered as having single RTN trap. The mechanism causing the disappearance of the third peak is discussed in Section 8.1.

Some of the 3-peak pixels show symmetric side peaks as in Figure 7, but some of the 3-peak pixels show asymmetric side peaks as in Figure 8. In yet some other cases, one of the side peaks disappears; only 2 peaks are observed as in Figure 9. All the examples in Figure 7, Figure 8 and Figure 9 are considered as pixels with a single RTN trap. More discussion on this is given in Section 8.1.

**Figure 10:**
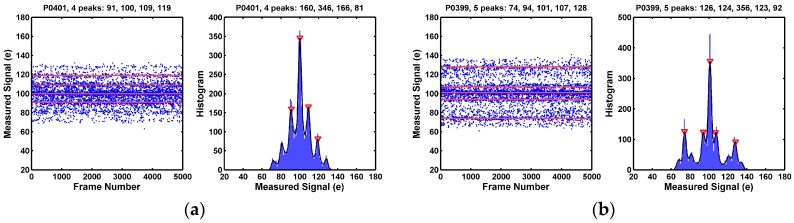

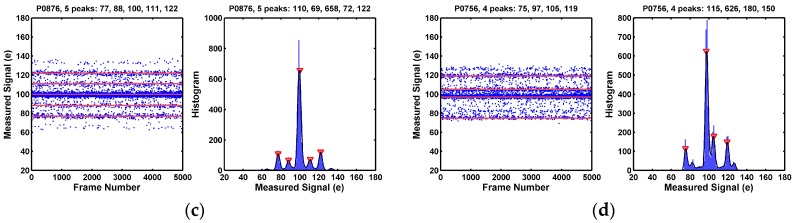
(**a**–**d**) Four example pixels showing more than 3 peaks. These pixels have more than one RTN traps. The pixel in (**a**) appears to show 7 peaks but the peak-finder program identified only 4; The pixel in (**b**) appears to show 9 peaks but the peak-finder program identified only 5. The total number of pixels showing more than 3 peaks is a small percentage of the total 8.3M population, in the order of 5~10 ppm.

A small percentage of pixels show more than 3 histogram peaks. They are pixels with multiple RTN traps. Some examples are given in Figure 10.

**Figure 11:**
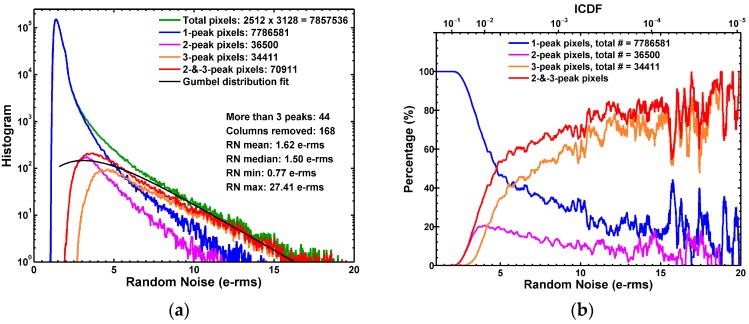
(**a**) The RN statistical distribution of all pixels, the 1-peak pixels, the 2-peak pixels, the 3-peak pixels, and the sum of the 2-peak and 3-peak pixels; (**b**) The 1-peak pixels, 2-peak pixels, 3-peak pixels, and the 2-or-3-peak pixels as the percentage of the total pixels. The key observation is that the 2-or-3-peak (single-trap) pixels are dominant in the high noise region with RN > 10 e−rms.

Most of the previous RTN studies in the literature involved either limited sample sizes or non-CIS processes. In this work we analyzed every pixel in an entire 8.3 MP array and classified them into 4 categories according to the number of observable peaks found in the 5000-frame signal histograms. A computer program was developed to sort the pixels automatically by identifying the local maximums in the histograms. Figure 11a shows the results of classification. The single-trap RTN pixels (the 2- and 3-peak pixels) account for about 1% of the total population. The pixels having multiple traps are very rare; only 44 are found in this sample.

Figure 11b shows the percentage of pixels with single RTN traps and the pixels without identifiable traps as a function of the noise amplitudes. The key finding is that the single-trap pixels represent 70 to 90% of the pixels in the high noise region (>10 e−rms). This is a solid piece of evidence linking the long tail of the noise distribution to the RTN.

## 5. On-Chip RTN Time Constant Extraction

On the microscopic level, the RTN originates from the random discrete emission and capture of a conduction electron by the electron trap. The probabilities of these discrete events can be calculated for any given number of transitions as a function of time [44]. Between the first and the second sampling phase of the CDS, if an odd number of transitions take place, the trap state would be changed and a disturbance of the signal contributes to the RTN. On the other hand, if there is an even number of transitions, the trap would remain in the same state; thus no noise is generated. The RTN as a function of the time difference between the first and the second sampling can be calculated by summing up the contributions from all the possible odd number of transitions [44].

Alternatively, on the macroscopic level, the probability of trap occupancy (PTO) may be considered as a smooth function of time P(t). The change rate of the PTO is the capture rate subtracted by the emission rate. This is described by the following first order differential equation:

(1) $$\frac{dP\left(t\right)}{dt}=\frac{1-P\left(t\right)}{\tau_{c}}-\frac{P\left(t\right)}{\tau_{e}},$$

where $\tau_{e}$ and $\tau_{c}$ are the emission and capture time constant of the trap. The general solution of above equation can be readily written as:

(2) $$P\left(t\right)=\frac{\tau_{e}}{\tau_{e}+\tau_{c}}+\left(P\left(0\right)-\frac{\tau_{e}}{\tau_{e}+\tau_{c}}\right)e^{-\frac{t}{\tau_{s}}};\;\tau_{s}≝\frac{\tau_{e}\tau_{c}}{\tau_{e}+\tau_{c}}.$$

From Equation (2) it is clear that, when the steady state is reached, the percentage of the RTN traps in the empty state is $\tau_{c}/\left(\tau_{e}+\tau_{c}\right)$ and the percentage in the occupied state is $\tau_{e}/\left(\tau_{e}+\tau_{c}\right)$. Moreover, the solution implies that the probability of an initially empty trap at t=0 becoming occupied after time t is $\left(\tau_{e}/\left(\tau_{e}+\tau_{c}\right)\right)\left(1-e^{-t/\tau_{s}}\right)$; vise versa, the probability of an initially occupied trap becoming empty after time t is $\left(\tau_{e}/\left(\tau_{e}+\tau_{c}\right)\right)\left(1-e^{-t/\tau_{s}}\right)$ [44,45]. A noise of magnitude ∆V is generated either when an empty trap changes to occupied or when an occupied trap changes to empty. Therefore the total noise power can be calculated as the sum of these two terms:

(3) $$n_{RTN}{\left(t\right)}^{2}={\left(∆V\right)}^{2}\left(\frac{\tau_{c}}{\tau_{e}+\tau_{c}}\right)\left(\frac{\tau_{e}}{\tau_{e}+\tau_{c}}\left(1-e^{-t/\tau_{s}}\right)\right)+{\left(∆V\right)}^{2}\left(\frac{\tau_{e}}{\tau_{e}+\tau_{c}}\right)\left(\frac{\tau_{c}}{\tau_{e}+\tau_{c}}\left(1-e^{-t/\tau_{s}}\right)\right),$$

which leads to the RMS noise as a function of time [44,45]:

(4) $$n_{RTN}\left(t\right)=\frac{\sqrt{2\tau_{e}\tau_{c}}}{\tau_{e}+\tau_{c}}\left(∆V\right)\sqrt{1-e^{-t/\tau_{s}}}.$$

**Figure 12:**
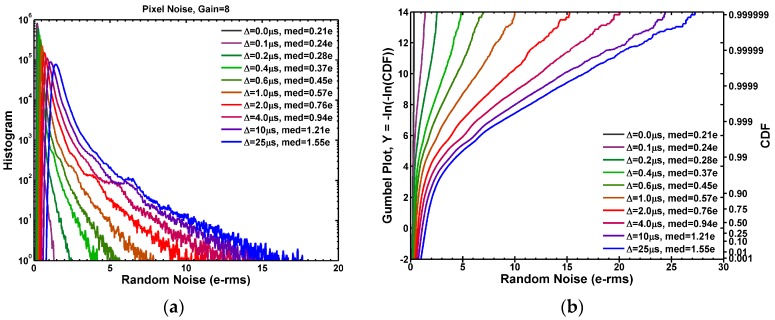
(**a**) The family of RN histograms when the double sampling time difference Δ*t* is gradually increased from 0 to 25 μs; (**b**) The Gumbel plots of the family of the CDF (cumulative distribution function) curves, which are approximately linear in the high noise region with RN > 10 e−rms.

Based on above formula, we developed a new method of on-chip RTN time constant extraction using the double sampling circuits. In this design, the time difference ∆*t* between the double sampling can be programmed from 0 to 25 μs in 0.1 μs steps while turning off the TG in dark RN measurements. The measured family of RN histograms indexed by ∆*t* is shown in Figure 12a.

When ∆*t* is set to 0 μs, the two samplings (SHR and SHS) are essentially correlated with respect to the SF noises; therefore, the SF RTN is completely cancelled and the median RN is as low as 0.21 e−rms. When ∆*t* is gradually increased towards 25 μs step by step, the two samplings become increasingly uncorrelated. The median RN increases to 1.55 e−rms at ∆*t* = 25 μs. It proves that the overall RN of this chip is primarily from the SF. The family of RN histograms can be expressed in a Gumbel plot in Figure 12b based on the Gumbel distribution [49] with the following probability density function (PDF) and the cumulative distribution function (CDF):

(5) $$PDF_{Gumbel}\left(x\right)=exp\left(-z-e^{-z}\right)/\beta ;\;CDF_{Gumbel}\left(x\right)=exp\left(-e^{-z}\right);\;z≝\left(x-\mu \right)/\beta .$$

where *x* is the random noise variable, *μ* is the mean, and the standard deviation is $\beta \pi /\sqrt{6}$. The RN long tails in Figure 12b clearly show the characteristics of the Gumbel distribution:

(6) $$-ln\left(-ln\left(CDF_{meas}\right)\right)\sim \left(x-\mu \right)/\beta .$$

**Figure 13:**
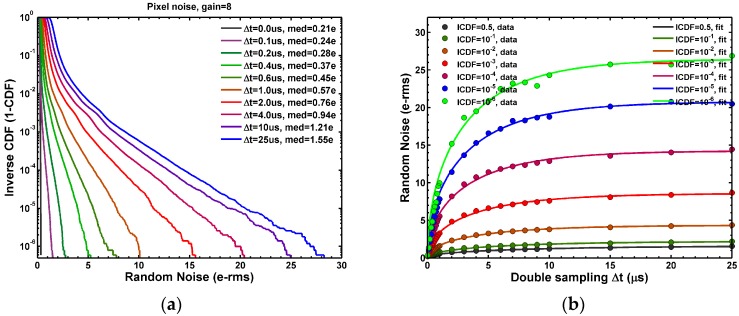
(**a**) The family of the ICDF (inverse CDF) curves of the RN distribution when Δ*t* is gradually increased from 0 to 25 μs; (**b**) The family of the constant-ICDF contours at ICDF = 0.5, 10^−1^, 10^−2^, 10^−3^, 10^−4^, 10^−5^, and 10^−6^, respectively. The contours were fit to the analytical Formula (4).

Another convenient way of expressing the data is to plot the logarithm of the inverse CDF (ICDF, or 1 − CDF) as in Figure 13a. Again the ICDF curves exhibit the similar linear dependence on RN in the long tail region with an opposite sign:

(7) $$ln\left(ICDF_{meas}\right)=ln\left(1-CDF_{meas}\right)\sim ln\left(-ln\left(CDF_{meas}\right)\right)\sim -\left(x-\mu \right)/\beta .$$

The constant ICDF contours are plotted in Figure 13b for ICDF at 0.5 (median), 10^−1^, 10^−2^, 10^−3^, 10^−4^, 10^−5^, and 10^−6^, respectively. The contours represent the RN evolution from the *correlated* double sampling at ∆*t* = 0 to the *uncorrelated* double sampling at ∆*t* = 25 μs with reference to the SF noises. All the constant ICDF contours in Figure 13b can be fit reasonably well by Equation (4) with a common characteristic time constant of 5.3 μs.

**Figure 14:**
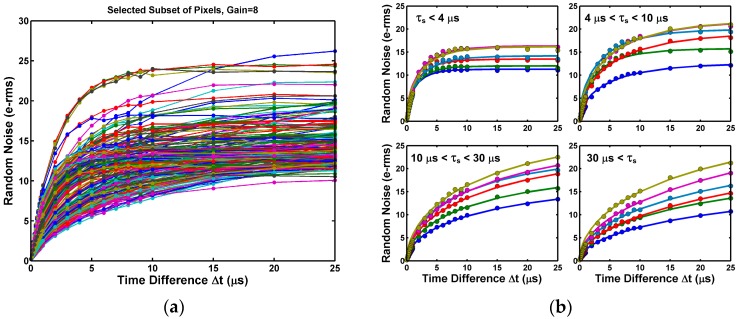
(**a**) The RN evolution from Δ*t* = 0 (correlated double sampling) to Δ*t* = 25 μs (uncorrelated double sampling) for a selected subset of 200 pixels; (**b**) Examples of pixels groups with various ranges of time constants from 1 μs to 500 μs.

More importantly, we found that the time constant *τ_s_* of each individual pixel could also be practically extracted by fitting Equation (4) to the measured RN as a function of ∆*t*. Figure 14a illustrates that the time dependence behavior varies widely from pixel to pixel. Figure 14b shows the extraction results of various pixels in different time constant ranges.

**Figure 15:**
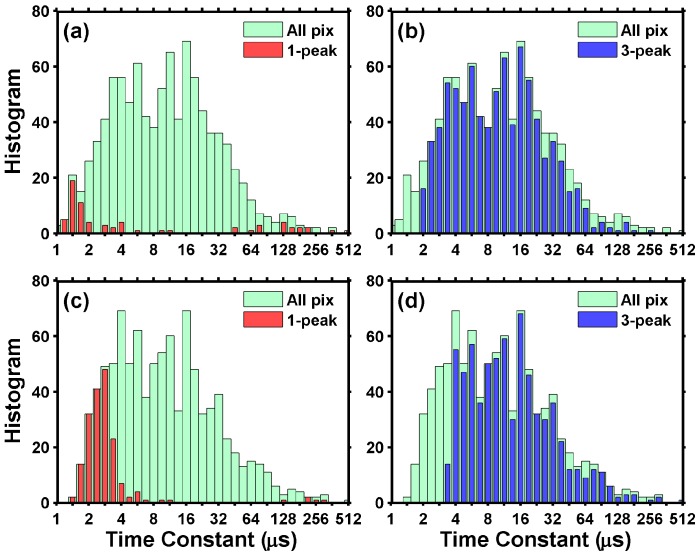
(**a**,**b**) The extracted RTN time constants distribution under 1X gain for the noisiest 1000 pixels; (**c**,**d**) The extracted RTN time constants under 8X gain for the noisiest 1000 pixels. All of the time constants fall within the range from 1 μs to 500 μs. Among the noisiest 1000 pixels, 80% of them show 3 peaks, and 15% show 1 peak. The time constant of the 1 peak pixels tend to be shorter than the 3 peak pixels.

Figure 15a,b show the *τ_s_* distribution of the noisiest 1000 pixels measured at gain 1X and Figure 15c,d are for gain 8X. The majority of the time constants fall within the range from 1 μs to 500 μs. We noted that the time constants longer than 100 μs might not be accurately extracted due to the limited range of curve fitting (25 μs). On the other hand, the time constants shorter than 5 μs are affected by the finite circuit bandwidth and settling time, to be discussed in the next section.

## 6. Circuit Settling Time and RTN Time Constant Extraction

Among the noisiest 1000 pixels, about 80% show 3 peaks and about 15% show 1 peak. Interestingly, Figure 15 shows that the 1-peak pixels tend to have shorter time constants, suggesting that some of the 1-peak pixels actually have 3 peaks (i.e., single-trap) but cannot be resolved due to the circuit bandwidth limitation.

**Figure 16:**
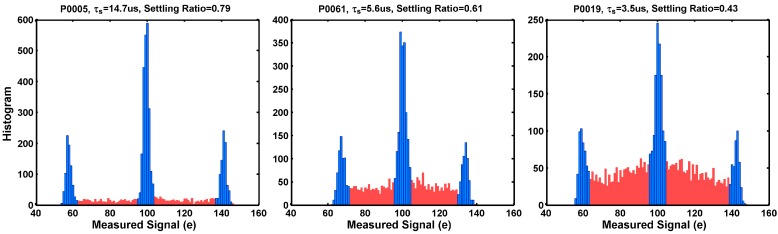
Due to the finite circuit settling time and the non-negligible RTN transition time, some of the data points fall into the neighborhood of the well-defined discrete levels (blue color) and some of the data points are in-between the discrete levels (red color). The settling ratio R is defined as the ratio of the settled data points versus the total data points. Three examples of pixels show the settling ratios of 0.79, 0.61, and 0.43, respectively, from left to right.

The simplified Equation (4) is based on an ideal assumption that the single RTN trap is either in an occupied state or an empty state, not anywhere in between. However, in the real circuits, the transition time between the occupied and the empty states are always finite because of the finite circuit response time and the bandwidth. Inevitably, the unsettled signal values between the two well-defined discrete states may also be captured during the measurement. In Figure 16, the settled data points are highlighted in blue, and the unsettled data points are in red color.

To analyze this quantitatively, we define a “settling ratio” for the 3-peak histogram as the ratio of the number of the settled points (the blue area) vs. the total sample size (the sum of the blue and the red area). For the example pixels in Figure 16a–c, the settling ratios are 0.79, 0.61, and 0.43, respectively. It can be intuitively expected that the settling ratio depends on the time constants of both the circuits and the RTN traps.

**Figure 17:**
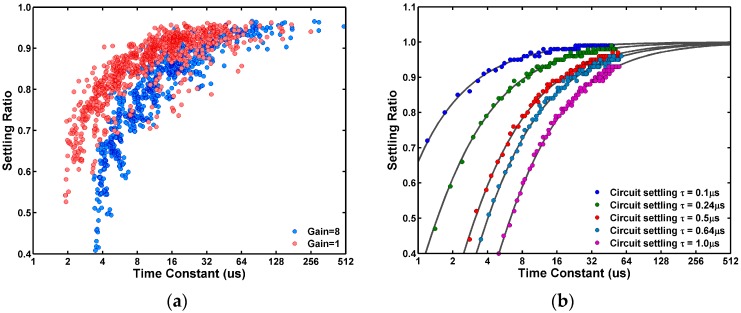
(**a**) The measured 3-peak pixels settling ratios versus the RTN time constants for the cases of 1X and 8X gains. At 1X gain the settling ratios are higher than those at 8X gain because of the circuit settling time at 1X gain is shorter than that at 8X gain; (**b**) The MATLAB RTN behavior simulation results approximately reproduce the observed trends in the measured data.

The settling ratios are plotted against the RTN time constants for the 1000 noisiest pixels in Figure 17a, under the 1X and 8X amplifier gains. The settling ratios approach 100% for pixels with longer time constants, but decrease sharply for pixels with shorter time constants. For the same pixel with a fixed time constant, the settling ratio is higher at 1X gain than that at 8X gain. Because the column amplifier bandwidth, determined by the ratio of the feedback capacitance C2 and the sampling capacitance C1, is higher at 1X gain than that at 8X gain.

**Figure 18:**
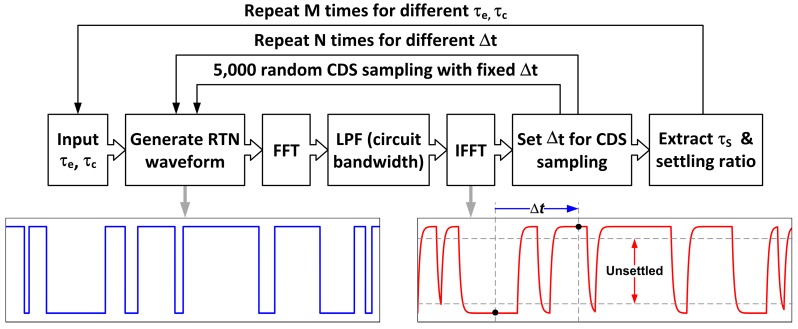
The RTN behavior model and simulation flow in MATLAB.

To further understand the data, we developed a RTN behavior model in MATLAB to simulate the settling ratio dependence on the time constant. The simulation flow diagram is explained in Figure 18. First, we generate an ideal random telegraphic waveform for a single-trap RTN for a given pair of emission and capture time constants with a large number of emission-capture transitions. Second, the time-domain waveform is transformed into the frequency domain using the FFT, multiplied by a low-pass filter corresponding to the circuit time constant, and transformed back to the time domain using the inverse FFT. Then, the periodically sampled RTN histogram and the settling ratio can be calculated from the resulting telegraphic waveform. The simulation results are graphed in Figure 17b. Evidently, the data are reasonably reproduced by the RTN behavior model, where the circuit time constants at 1X and 8X gains are approximately 0.24 μs and 0.64 μs, respectively, from circuit simulation.

## 7. The RN and RTN Dependence on Temperature

**Figure 19:**
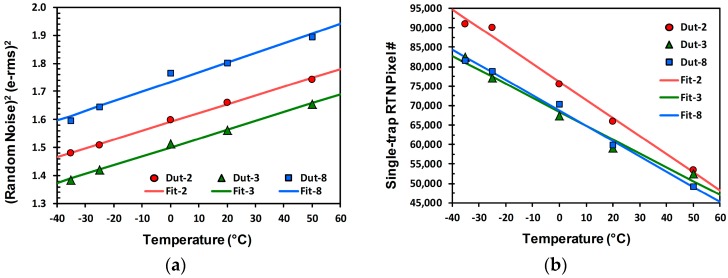
(**a**) The squares of the RN medians measured at the 8X gain are linearly dependent on the temperature, suggesting that they are dominated by the thermal noises; (**b**) The number of pixels with single RTN trap, i.e., the 2- and 3-peak pixels, increases as the temperature decreases. Because the longer time constants at lower temperature makes more pixels with RTN histogram peaks identifiable.

As discussed in Section 4, the lower noise region of the RN distribution is dominated by pixels without RTN traps, which accounts for more than 90% of the total population. While the higher noise region of the RN distribution is dominated by RTN pixels, accounting for less than 1% of the population. As a result, the temperature dependence of the lower noise and the higher noise regions behave differently. In Figure 19a, we can see that the squares of the RN medians are linearly proportional to the temperature, which is the characteristic behavior of the thermal noise. This is consistent with previous conclusions that median noises are dominated by the pixels without RTN traps. The noise sources are primarily the thermal noises and the flicker noises. And the statistical distribution is close to the Gaussian distribution.

On the contrary, the pixels with RTN traps behave differently as temperature changes. In Section 6, we proved that the identified number of single-trap RTN pixels is related to the readout circuit time constants. Figure 19b shows that the number of single-trap RTN pixels increases as the temperature decreases. This is because the RTN time constants increase significantly as the temperature is lowered, while the circuit time constants changes are nearly negligible. Therefore, the RTN histogram settling ratios increase at lower temperatures. The number of identifiable RTN pixels increases, because for many pixels the RTN discrete levels cannot be observed at higher temperature but can be observed at lower temperatures.

**Figure 20:**
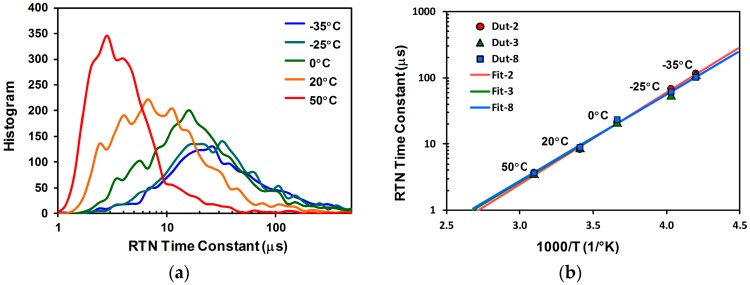
(**a**) The distributions of the RTN characteristic time constants at different temperatures. The RTN time constants are extracted from the 1000 noisiest pixels of 3 samples; (**b**) The Arrhenius plots of the medians of the RTN time constants for 3 samples.

The distributions of the characteristic RTN time constants from −35 °C to 50 °C are shown in Figure 20a, for a total of 3000 noisiest pixels from 3 samples. It is clear that the time constants vary widely from sample to sample, but systematically move towards higher values at lower temperatures. Moreover, the Arrhenius plot of Figure 20b shows that the medians of the time constants are linearly proportional to the reciprocals of the absolute temperatures, following a well-defined Arrhenius equation with an effective activation energy $E_{a}\sim 0.11\;\mathrm{eV}$:

(8) $$\tau_{s}\left(T\right)=\tau_{s}\left(T_{o}\right)\;exp\left(\frac{E_{a}}{k_{B}}\left(\frac{1}{T}-\frac{1}{T_{0}}\right)\right),$$

It should be noted that the individual emission and capture time constants may have very different temperature dependence [37,40,41,43]. In this report we only extracted an effective activation energy for the characteristic time constant $\tau_{s}=\tau_{e}\tau_{c}/\left(\tau_{e}+\tau_{c}\right)$.

## 8. Discussion on Several Subtle or Unresolved Issues

In this section, we address several subtle issues that are not fully resolved or fully understood. These will be the subjects for future investigation.

### 8.1. The Symmetric and Asymmetric 3 Peak RTN Pixels

**Figure 21:**
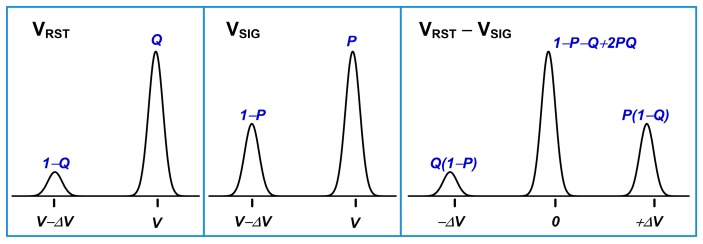
A hypothetical noise distribution before and after CDS. The reset noise V_RST_ is sampled first (the leftmost panel) and the signal voltage V_SIG_ is sampled second (the middle panel). The 2 results are subtracted by CDS operation (the rightmost panel). Assuming that the probability of trap occupancy is *Q* at the first sampling and *P* at the second sampling, the probabilities of the 3 peaks after CDS can be calculated as in the rightmost panel. If *P* = *Q* the 2 side peaks would be symmetric. If *Q* ≠ *P* we would observe 2 asymmetric side peaks. In the case of *Q* = 0, only 2 peaks are observable.

The observed 2-peak pixels and the 3-peak pixels, with either 2 symmetric or asymmetric side peaks, are all considered as having a single RTN trap. The variation in the behavior can be explained by the PTO in the two phases of the CDS and the device operation conditions. Figure 21 shows the hypothetical signal distribution without and with the CDS subtraction. The reset noise V_RST_ is sampled in the first phase of CDS and the signal voltage V_SIG_ is sampled in the second phase. Suppose there is a RTN trap in the SF, the signal distribution would show 2 discrete levels, corresponding to the empty and the occupied trap states, where *P* and *Q* are the PTO during the first and the second sampling phase, respectively. The probabilities of the traps being empty would be 1 − *P* and 1 − *Q*. The illustration is hypothetical because the KTC noises are not cancelled in each individual phase, but only cancelled after the CDS subtraction. Since the KTC noises are much higher than all the other noises combined, the full-width at half-maximum (FWHM) of the distribution would be much broader. For this chip, the KTC noise is estimated to be 14 e−rms while the real measured noise is only 1.55 e−rms with the CDS.

The probabilities of the 3 peaks after the CDS can then be calculated straightforwardly if the 2 sampling events are assumed statistically independent. With this in mind, we can see clearly that if *P* = *Q*, the resulting 2 side peaks would be symmetric; vice versa, if *P* ≠ *Q* the 2 side peaks would be asymmetric. In the extreme situation when *Q* = 0, the lower side peak would disappear completely. All above three cases are clearly observed from the data in Figure 7, Figure 8 and Figure 9.

**Figure 22:**
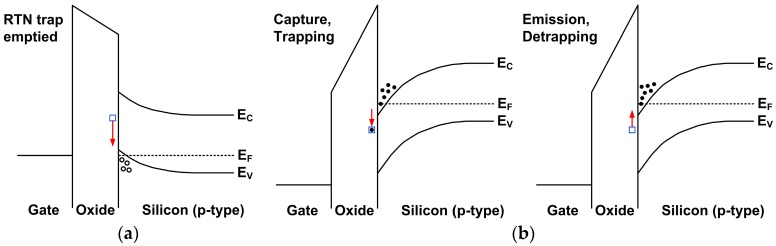
(**a**) Band diagram for a NMOS SF under the accumulation condition when it is turned OFF. The Si surface is populated with high concentration of holes. The electron trap is likely to be empty; (**b**) The band diagram for a NMOS SF under the inversion condition when it is turned ON. The Si surface is populated with high concentration of electrons. The capture and emission events happen randomly, governed by the capture and emission time constants, and may reach a steady state after certain time.

The reason causing the asymmetry can be explained by the SF operation conditions and the energy band diagrams in Figure 22. For the pixel without the row-select device, the SF serves the purpose of row selection. Since all the SFs on the same column share the same column signal line, only 1 SF is allowed to be active at any given time. That is the time when the selected row is being reset or read out. During the time of photo carrier integration, the SF needs to be disabled by pulling low the FD voltage to below the threshold voltage of the SF as shown in Figure 2 timing diagram. When the SF is turned OFF, the channel is in the accumulation condition (Figure 22a) and populated by high concentration of holes. Therefore, the electron trap is likely to be empty. When the SF is turned ON during the CDS readout, the channel is in the strong inversion condition (Figure 22b) with a high concentration of electrons near the interface of Si and gate oxide. The electron trap interacts with the conducting electrons in the channel thru the process of random emission and capture. It takes a finite amount of time for the trapping and de-trapping to reach a stead state, depending on the time constants. For some single trap pixels, the steady state is reached quickly such that the PTO is the same for the first and the second sampling, leading to 2 symmetric side peaks. For some other pixels, the steady state is not reached fast enough, and the PTO in the first sampling (*Q*) is lower than that in the second sampling (*P*). Thus the 2 side peaks appear to be asymmetric as in Figure 8. In the third case, the PTO in the first sampling is nearly zero; then only 1 side peak is observable. The reason why some pixels behave differently from the others may have to do with the trap energies relative to the Fermi levels of the channel electrons and the physical locations of the traps inside the gate oxide. These are not fully understood and require further investigation.

In the literature, a switched biasing technique was proposed to reduce low-frequency noises and RTN [50,51,52]. Our data appear to show that the technique may not be universally effective for all RTN traps. It may be strongly dependent on the energy levels and the physical locations of the traps.

### 8.2. The Effects of Column RTN

**Figure 23:**
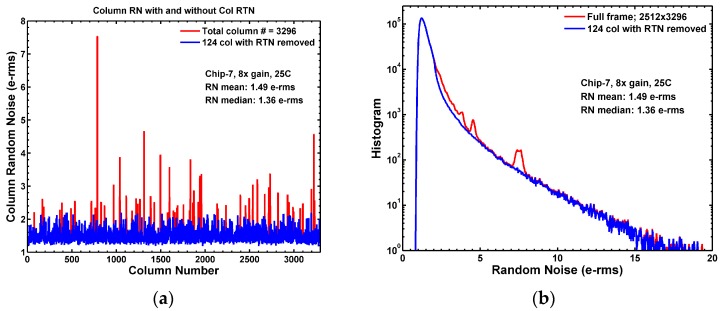
Column RN and pixel RN. (**a**) The column RN is defined as the average of the RMS of pixel RN for all pixels on the same column. The columns showing high noises are suspected to have RTN traps in the column bias devices. An empirical threshold was set such that these RTN columns are excluded in the pixel noise histogram calculation; (**b**) The noise histograms without deleting the RTN columns (red) show bumps and irregularities because a RTN trap in the column circuit boosts up the noises of all the pixels on the same column. The curve becomes smoother if the RTN columns are excluded (blue). This chip is different from the one shown in Figure 12 and Figure 13.

It should be pointed out that, not only some of the pixels, but also some of the column bias transistors may have RTN traps. By simply inspecting the full frame RN histogram, it is difficult to distinguish the column RTN from the pixel RTN. An example of the column RN of 3296 columns is shown in Figure 23a, where the column RN is defined as the column average of the pixel RN for all pixels on the same column. Most of the columns have column RN close to the frame average. However, some columns show exceptionally higher column RN. Empirically, these columns with higher column RN are considered as the columns with RTN traps in the column circuits, specifically in the column current source NMOS that drives the entire column signal line. A column circuit with RTN traps would boost up the pixel RN for all 2512 pixels on the same column. The effect shows up as irregularities and the unnatural humps on the noise histogram, as the red curve in Figure 23b. In our data analysis, since we focus on the RN statistics of the pixels, we decided to exclude the columns with high probabilities of RTN traps from the pixel statistical calculation. In the example of Figure 23a, 142 out of 3296 such high RN columns are excluded. An empirical RN threshold was set to single out these columns. The blue curve in Figure 23b shows that the irregularities and the humps in the histograms are visibly removed. This may not be a rigorous approach, but it is effective to untangle the column RTN and the pixel RTN.

In this chip, the column current source is a W = 1 μm, L = 1 μm NMOS. The relatively small size of this device makes it vulnerable to RTN traps. It was well-reported that increasing the device W and L reduced the impact of RTN. For small pixels, there is probably not much room to use larger SF devices. But for the front-end column circuits such as the column amplifier or the column comparator, it is, in general, recommended to use larger devices wherever possible to minimize the potential impact of column RTN. The result implies that, for 3D stacked CIS, minimizing the RTN of the circuits on the ASIC die is as important as minimizing the RTN of pixels on the CIS die.

### 8.3. The Pixels That Are Difficult to Classify

**Figure 24:**
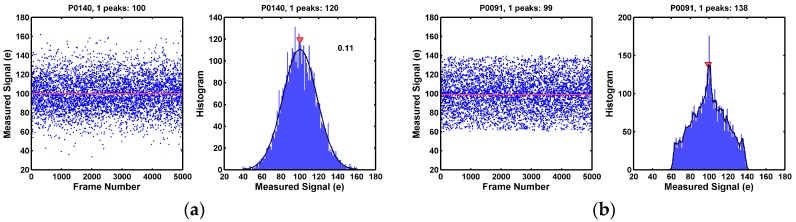
(**a**,**b**) Two examples pixels showing one peak with relatively high RMS noises. It is not clear whether these pixels have no RTN traps, one trap, or many traps. We speculate that at least some of these pixels have one or many RTN traps with closely spaced discrete levels, or with very short time constants, such that the readout circuit cannot separate the peaks.

The classification of pixels according the observed noise histogram peaks sometimes is not as straightforward as we might expect. First, there are always glitches or unevenness in the measurement, even 5000 frames of the data were taken for the pixel-by-pixel histogram calculation. Some level of low pass filtering on the histogram is necessary in order to root out the glitches from the real RTN peaks. Second, an ad hoc threshold has to be set in order to identify a local maximal point as a RTN peak. If the threshold is set too high, some small peaks will be missed. If the threshold is set too low, some noises or glitches in the data will be misidentified. Furthermore, there are cases in the border area between two categories. For instance, some of the one-peak pixels in Figure 24 show higher noises and larger standard deviations than other low-noise one-peak pixels. It is speculated that these pixels may have a single RTN trap or even multiple traps, but the discrete levels simply could not be resolved due to the circuit limitation. Indeed, when the same chip is measured at a lower temperature, the RTN time constants become longer and we did observe that some (but not all) 1-peak pixels start to behave more like three-peak, or more-than-three-peak pixels. We also observed that the temperature dependence of the RTN time constant varies very widely from pixel to pixel. The study is still a work in progress.

## 9. Conclusions

A statistical analysis of the RTN of a 1.1 μm pitch, 8.3 Mpixel CIS fabricated in a 45 nm BSI technology is presented in this paper. To summarize, we have:

- Established a method of noise source decomposition;
- Demonstrated that the RTN was mainly from the pixel SF;
- Proved that the long tail of the noise distribution was dominated by RTN;
- Systematically sorted the 8.3 MP into four groups according to the RTN peaks;
- Derived an analytic formula to explain the double sampling time dependence;
- Developed an on-chip RTN time constant extraction method;
- Performed pixel-by-pixel time constant extraction for 1000 noisiest pixels;
- Accounted for the settling ratio dependence on the single-trap RTN time constant and the readout circuit settling time constant by simulation.

Some of the unresolved issues are discussed in Section 8, which should be the subjects of further studies. In addition, the comparison of RTN performance across technology nodes and the RTN trend of device scaling are important topics for future investigation.

## Figures and Tables

**Table 1:** The 8.3 MP CMOS Image Sensor (CIS) physical and performance parameters.

Physical Parameter	Value	Performance Parameter	Value
Technology	45 nm BSI	Full well capacity	~6000 e−
Array resolution	3296 × 2512	Read noise (@ 8X)	<2 e−rms
Pixel cell size	1.1 μm × 1.1 μm	Dark current (@ 60 °C)	<2 e−/s
Pixel sharing type	2 × 2	Photo response non-uniformity (PRNU)	<1%
Transistors per cell	1.5	FD capacitance	~1.34 fF
Source follower W/L	0.2 μm/0.8 μm	Conversion gain	~ 20 μV/e−
Column biase W/L	1 μm/1 μm	System conversion factor (@ 8X)	~0.153 e−/DN

**Table 2:** The four operation modes and the corresponding temporal noise sources.

Noise Source vs. Test Mode	Photon Shot Noise	Reset KTC Noise	Pixel Source Follower	Column Current Source	Column Amplifier	Rest of Circuits
Pixel-in, Δ*t* = 25 μs	N/A	Cancelled	Yes	Yes	Yes	Yes
Test-in, Δ*t* = 25 μs	N/A	Bypassed	Bypassed	Bypassed	Yes	Yes
Pixel-in, Δ*t* = 0 μs	N/A	Cancelled	Cancelled	Cancelled	Cancelled	Yes
Test-in, Δ*t* = 0 μs	N/A	Bypassed	Bypassed	Bypassed	Cancelled	Yes

## Abbreviations

The following abbreviations are used in this manuscript:

ADC	analog to digital converter
BSI	backside illumination
CCD	charge coupled device
CDF	cumulative distribution function
CDS	correlated double sampling
CIS	CMOS image sensor
CMOS	complementary metal oxide semiconductor
CSL	column select
DN	digital number
DRAM	dynamic random access memory
FD	floating diffusion
FPGA	field programmable gate array
FPN	fixed pattern noise
FWC	full well capacity
FWHM	full width half maximum
ICDF	inverse cumulative distribution function
KTC noise	the thermal noise on a capacitor in the form of $\sqrt{k_{B}T/C}$
NAND Flash	a nonvolatile memory based on the NAND logic gate
NMOS	*n*-channel MOSFET
MATLAB	a proprietary programming language and software package by MathWorks
MOSFET	metal oxide semiconductor field effect transistor
PD	photodiode
PDF	probability density function
PPD	pinned photodiode
ppm	part per million
PTC	photon transfer curve
PTO	probability of trap occupancy
PRNU	photo response non-uniformity
RN	random noise
RMS	root mean square
RTN	random telegraph noise
S/H	sample and hold
SF	source follower
SRAM	static random access memory
STI	shallow trench isolation
TG	transfer gate

## References

[1] Kirton M.J., Uren M.J. (1989). Noise in solid-state microstructures: A new perspective on individual defects, interface states and low-frequency (1/*f*) noise. Adv. Phys..

[2] Hung K.K., Kuo P.K., Hu C., Cheng Y.C. (1989). An automated system for measurement of random telegraph noise in MOSFE. IEEE Trans. Electron Devices.

[3] Shi Z., Mieville J.-P., Dutoit M. (1994). Random telegraph signals in deep submicron n-MOSFET’s. IEEE Trans. Electron Devices.

[4] Ghibaudo G., Boutchacha T. (2002). Electrical noise and RTS fluctuations in advanced CMOS devices. Microelectron. Reliab..

[5] Simoen E., Claeys C. (2002). Random telegraph signal: A local probe for single point defect studies in solid-state devices. Mater. Sci. Eng..

[6] Simoen E., Kaczer B., Toledano-Luque M., Claeys C. (2011). Random telegraph noise: From a device physicist’s dream to a designer’s nightmare. Electrochem. Soc. Trans..

[7] Walczak K., Reinecke T.L. (2011). Scaling rules for telegraph noise. IEEE Trans. Nanotechnol..

[8] Ackerson K., Musante C., Gambino J., Ellis-Monaghan J., Maynard D., Rassel R.J., Ogg K., Jaffe M. Characterization of “blinking pixels” in CMOS image sensors. Proceedings of the 2008 IEEE Advanced Semiconductor Manufacturing Conference (ASMC).

[9] Peters I., Bogaart E., Manoury E.-J., Mierop A., Bosiers J. Analysis of blinking pixels in CCD imagers with and without surface pinning. Proceedings of the 2011 IEEE International Image Sensor Workshop (IISW).

[10] Mori Y., Ohyu K., Okonogi K., Yamada R.-I. The origin of variable retention time in DRAM. Proceedings of the 2005 IEEE International Electron Devices Meeting (IEDM).

[11] Oh B., Cho H.-J., Kim H., Son Y., Kang T., Park S., Jang S., Lee J.-H., Shin H. (2011). Characterization of an oxide trap leading to random telegraph noise in gate-induced drain leakage current of DRAM cell transistors. IEEE Trans. Electron Devices.

[12] Takeuchi K., Nagumo T., Hase T. Comprehensive SRAM design methodology for RTN reliability. Proceedings of the 2011 IEEE Symposium on VLSI Circuits.

[13] Toh S.O., Liu T.-J.K., Nikolić B. Impact of random telegraph signaling noise on SRAM stability. Proceedings of the 2011 IEEE Symposium on VLSI Circuits.

[14] Miki H., Osabe T., Tega N., Kotabe A., Kurata H., Tokami K., Ikeda Y., Kamohara S., Yamada R. Quantitative analysis of random telegraph signals as fluctuations of threshold voltages in scaled flash memory cells. Proceedings of the 2007 IEEE International Reliability Physics Symposium (IRPS).

[15] Kang D., Lee S., Park H.-M., Lee D.-J., Kim J., Seo J., Lee C., Song C., Lee C.-S., Shin H. A new approach of NAND flash cell trap analysis using RTN characteristics. Proceedings of the 2011 IEEE Symposium on VLSI Technology.

[16] Matsumoto T., Kobayashi K., Onodera H. Impact of random telegraph noise on CMOS logic delay uncertainty under low voltage operation. Proceedings of the 2012 IEEE International Electron Devices Meeting (IEDM).

[17] Luo M., Wang R., Guo S., Wang J., Zou J., Huang R. (2015). Impacts of random telegraph noise (RTN) on digital circuits. IEEE Trans. Electron Devices.

[18] Tega N., Miki H., Ren Z., D’Emic C.P., Zhu Y., Frank D.J., Guillorn M.A., Park D.G., Haensch W., Torii K. Impact of HK/MG stacks and future device scaling on RTN. Proceedings of the 2011 IEEE International Reliability Physics Symposium (IRPS).

[19] Dongaonkar S., Giles M.D., Kornfeld A., Grossnickle B., Yoon J. Random telegraph noise (RTN) in 14 nm logic technology: High volume data extraction and analysis. Proceedings of the 2016 IEEE Symposium on VLSI Technology.

[20] Findlater K.M., Vaillant J.M., Baxter D.J., Augier C., Herault D., Henderson R.K., Hurwitz J.E.D., Grant L.A., Volle J.M. (2004). Source follower noise limitations in CMOS active pixel sensors. Proc. SPIE.

[21] Kolhatkar J.S., Hoekstra E., Salm C., van der Wel A.P., Klumperink E.A.M., Schmitz J., Wallinga H. Modeling or RTS noise in MOSFETs under steady-state and large-signal excitation. Proceedings of the 2004 IEEE International Electron Devices Meeting (IEDM).

[22] Hoekstra E. Large signal excitation measurement techniques for RTS noise in MOSFETs. Proceedings of the 2005 IEEE EUROCON.

[23] Leyris C., Vildeuil J.C., Roy F., Martinez F., Valenza M., Hoffmann A. Response of correlated double sampling CMOS imager circuit to random telegraph signal noise. Proceedings of the 2006 IEEE International Caribbean Conference on Devices, Circuits and Systems (ICCDCS).

[24] Janesick J., Andrews J.T., Elliott T. (2006). Fundamental performance differences between CMOS and CCD imagers; Part I. Proc. SPIE.

[25] Janesick J., Pinter J., Potter R., Elliott T., Andrews J., Tower J., Grygon M., Keller D. (2010). Fundamental performance differences between CMOS and CCD imagers; Part IV. Proc. SPIE.

[26] Wang X., Rao P.R., Mierop A., Theuwissen A.J.P. Random telegraph signal in CIS pixels. Proceedings of the 2006 IEEE International Electron Devices Meeting (IEDM).

[27] Wang X., Snoeij M.F., Rao P.R., Mierop A., Theuwissen A.J.P. A CMOS image sensor with a buried-channel source follower. Proceedings of the 2008 IEEE International Solid-State Circuits Conference (ISSCC).

[28] Chen Y., Wang X., Mierop A., Theuwissen A.J.P. (2009). A CMOS image sensor with in-pixel buried-channel source follower and optimized row selector. IEEE Trans. Electron Devices.

[29] Martin-Gonthier P., Magnan P. Low-frequency noise impact on CMOS Image sensors. Proceedings of the 24th Conference on Design of Circuits and Integrated Systems (DCIS).

[30] Martin-Gonthier P., Magnan P. RTS noise impact in CIS readout circuit. Proceedings of the 2009 IEEE 16th International Conference on Electronics, Circuits and Systems (ICECS).

[31] Martin-Gonthier P., Havard E., Magnan P. (2010). Custom transistor layout design techniques for random telegraph signal noise reduction in CIS. Electron. Lett..

[32] Abe K., Sugawa S., Kuroda R., Watabe S., Miyamoto N., Teramoto A., Ohmi T., Kamata T., Shibusawa K. Analysis of source follower random telegraph signal using nMOS and pMOS array TEG. Proceedings of the 2007 IEEE International Image Sensor Workshop (IISW).

[33] Fujisawa T., Abe K., Watabe S., Miyamoto N., Teramoto A., Sugawa S., Ohmi T. (2010). Analysis of hundreds of time constant ratios and amplitudes of random telegraph signal with very large scale array test pattern. Jpn. J. Appl. Phys..

[34] Abe K., Teramoto A., Watabe S., Fujisawa T., Sugawa S., Kamata Y., Shibusawa K., Ohmi T. (2010). Experimental investigation of effect of channel doping concentration on random telegraph signal noise. Jpn. J. Appl. Phys..

[35] Yonezawa A., Teramoto A., Kuroda R., Suzuki H., Sugawa S., Ohmi T. Statistical analysis of random telegraph noise reduction effect by separating channel from the interface. Proceedings of the 2012 IEEE International Reliability Physics Symposium (IRPS).

[36] Kuroda R., Yonezawa A., Teramoto A., Li T.L., Tochigi Y., Sugawa S. (2013). A statistical evaluation of random telegraph noise of in-pixel source follower equivalent surface and buried channel transistors. IEEE Trans. Electron Devices.

[37] Obara T., Yonezawa A., Teramoto A., Kuroda R., Sugawa S., Ohmi T. (2014). Extraction of time constants ratio over nine orders of magnitude for understanding random telegraph noise in MOSFETs. Jpn. J. Appl. Phys..

[38] Obara T., Teramoto A., Yonezawa A., Kuroda R., Sugawa S., Ohmi T. Analyzing correlation between multiple traps in RTN characteristics. Proceedings of the 2014 IEEE International Reliability Physics Symposium (IRPS).

[39] Kuroda R., Teramoto A., Sugawa S. Random telegraph noise measurement and analysis based on arrayed test circuit toward high S/N CMOS image sensors. Proceedings of the 2016 IEEE 29th International Conference on Microelectronic Test Structures (ICMTS).

[40] Realov S., Shepard K.L. Random telegraph noise in 45-nm CMOS: Analysis using an on-chip test and measurement system. Proceedings of the 2010 IEEE International Electron Devices Meeting (IEDM).

[41] Nagumo T., Takeuchi K., Hase T., Hayashi Y. Statistical characterization of trap position, energy, amplitude and time constants by RTN measurement of multiple individual traps. Proceedings of the 2010 IEEE International Electron Devices Meeting (IEDM).

[42] Realov S., Shepard K.L. (2013). Analysis of random telegraph noise in 45-nm CMOS using on-chip characterization system. IEEE Trans. Electron Devices.

[43] Chen J., Higashi Y., Kato K., Mitani Y. Further understandings on random telegraph signal noise through comprehensive studies on large time constant variation and its strong correlations to thermal activation energies. Proceedings of the 2014 IEEE Symposium on VLSI Technology.

[44] Chao C.Y.-P., Tu H., Wu T., Chou K.-Y., Yeh S.-F., Hsueh F.-L. (2017). CMOS image sensor random telegraph noise time constant extraction from correlated to uncorrelated double sampling. IEEE J. Electron Devices Soc..

[45] Chao C.Y.-P., Tu H., Wu T., Chou K.-Y., Yeh S.-F., Hsueh F.-L. Random Telegraph Noise Pixel Classification and Time Constant Extraction for a 1.1 µm Pitch 8.3 MP CMOS Image Sensor. Proceedings of the 2017 IEEE International Image Sensor Workshop (IISW).

[46] Kuroda R., Teramoto A., Sugawa S. Impact of random telegraph noise with various time constants and number of states in CMOS image sensors. Proceedings of the 2017 IEEE International Image Sensor Workshop (IISW).

[47] Fossum E.R., Hondongwa D.B. (2014). A review of the pinned photodiode for CCD and CMOS image sensors. IEEE J. Electron Devices Soc..

[48] Janesick J. (2007). Photon Transfer: DN→λ.

[49] Walck C. (2007). Chapter 15 Extreme Value Distribution. Hand-Book on Statistical Distributions for Experimentalists.

[50] Martin S., Li G., Worley E., White J. Modeling the bias and scaling dependence of drain current fluctuations due to single carrier trapping in submicron MOSFET’s. Proceedings of the Digest of the IEEE 1996 54th Annual Device Research Conference (DRC).

[51] Snoeij M.F., van der Wel A.P., Theuwissen A.J.P., Huijsing J.H. The effect of switched-biasing on 1/*f* noise in CMOS imager front-ends. Proceedings of the 2005 IEEE Workshop on CCDs and Advanced Image Sensors.

[52] Martin-Gonthier P., Magnan P. (2011). Novel readout circuit architecture for CMOS image sensors minimizing RTS noise. IEEE Electron Device Lett..

